# COVID-19 Screening in the Pediatric Emergency Department

**DOI:** 10.7759/cureus.35731

**Published:** 2023-03-03

**Authors:** Deborah L Hammett, Claire Loiselle, Kathleen M Palmer, John M Loiselle, Magdy W Attia

**Affiliations:** 1 Pediatric Emergency Medicine, Nemours Children's Hospital, Wilmington, USA; 2 Biomedical Research, Nemours Children's Hospital, Wilmington, USA

**Keywords:** hospital-admission, emergency department, pediatrics, screening, sars-cov-2, covid-19

## Abstract

Background:Screening for COVID-19 infection in pediatrics is challenging as its clinical presentation may be asymptomatic or mimic other common childhood infections. We examined the use of a COVID-19 screening protocol (CSP) in the pediatric emergency department (PED) to determine the incidence of positive severe acute respiratory syndrome coronavirus 2 (SARS-CoV-2) polymerase chain reaction (PCR) tests in patients who are CSP+ and CSP-.

Methods: We conducted a retrospective cohort study of pediatric patients with SARS-CoV-2 testing completed in an urban tertiary care PED from November 1 to December 31, 2020. Demographics, CSP designation, test results, and disposition were compared. Statistical significance was determined using chi-square or a comparison of means. Sensitivity, specificity, negative predictive value (NPV), and positive predictive value (PPV) with 95% confidence intervals (CI) were calculated.

Results: A total of 1,613 patients had SARS-CoV-2 tests completed with 9.1% (N=147) having positive test results. Of 1,014 (62.9%) patients who were CSP+, 12.9% tested positive. Comparatively, 599 (37.1%) patients were CSP- with only 2.7% positive tests, p<0.0001. The sensitivity, specificity, NPV, and PPV of the CSP in all tested patients were 89.1%, 39.8%, 97.3%, and 12.9%, respectively. Of tested patients, 887 (55.0%) were admitted to the hospital and were more likely to be positive if CSP+, p≤0.001. Within the admitted group, 16.8% were admitted to the operating room, of whom 83.9% were CSP- with 4.0% testing positive for SARS-CoV-2.

Conclusions: COVID-19 screening in the pediatric population is a useful modality to risk stratify most patients presenting to the PED for the purpose of selective testing and guiding personal protective equipment use. This may be particularly useful in low-resource settings.

## Introduction

The rapid spread of the severe acute respiratory syndrome coronavirus 2 (SARS-CoV-2) virus and the potential for severe illness associated with infection led the World Health Organization to declare the COVID-19 outbreak a global pandemic in March 2020 [1]. In addition to developing treatment strategies to care for infected patients, healthcare providers and hospital systems developed approaches to limit transmission of the virus to protect both patients and frontline workers. Given that the emergency department (ED) serves as a key point of entry for patients to the hospital, many institutions developed adaptations to ED workflow to screen and isolate potential cases of COVID-19 [2-4]. Evidence in the adult literature suggests that screening patients with respiratory symptoms and proper use of personal protective equipment (PPE) has a high sensitivity, and resulting protective measures enabled one institution to achieve its goal of zero cases of SARS-CoV-2 through nosocomial transmission [5].

Compared with the adult population, COVID-19 infection affects fewer pediatric patients; however, the number of cases of COVID-19 in children has been noted by the Centers for Disease Control and Prevention (CDC) to be rising since March 2020 [6]. Children are now estimated to account for approximately 19% of all reported COVID infections [7]. The severe disease remains uncommon in pediatric cases of SARS-CoV-2 infection with fatality estimates ranging from 0.12% to 0.2% of cases [8,9]. Given that there are fewer cases of COVID-19 reported in children, little has been published on the utility of COVID-19 screening in pediatrics.

Fever, rhinorrhea, cough, sore throat, nausea, vomiting, diarrhea, fatigue, myalgias, and headache have all been identified as presenting symptoms of COVID-19 in children [10,11]. SARS-CoV-2 infection has even been associated with croup and stridor in some pediatric patients [12]. Additionally, children have been reported to be asymptomatic when infected with SARS-CoV-2 [13,14]. This poses an added level of complexity when attempting to identify and risk stratify COVID-19-infected pediatric patients.

The primary aim of this study was to evaluate the utility of COVID-19 screening in the pediatric emergency department (PED) by comparing the rate of positive SARS-CoV-2 tests in patients who screen “positive” with those who screen “negative” at ED triage. Secondary aims of the project include looking at the SARS-CoV-2 test positivity among COVID-19 screening protocol (CSP) positive (+) and CSP negative (-) groups within the subsets of patients admitted to the hospital, admitted to the operating room (OR), and discharged from the ED.

## Materials and methods

Study design

During the pandemic, the ED developed a database for the purpose of result follow-up and continuity of care for patients undergoing SARS-CoV-2 testing in the PED. This database was utilized to conduct a retrospective cohort study of pediatric patients with SARS-CoV-2 testing completed in the PED at an urban tertiary care children’s hospital in the United States with approximately 60,000 ED visits annually. The study population included patients ≤21 years of age with SARS-CoV-2 testing completed from November 1, 2020, to December 31, 2020. The study was approved by the institutional review board (Nemours Foundation, IORG IRB00000293) and deemed exempt from consent due to the retrospective nature of the investigation. Standard protocols were utilized to assure that patient confidentiality was protected.

Procedure

To guide PPE usage and to cohort potential cases of COVID-19, the hospital independently made the decision to adopt the CSP in the PED during the pandemic. The hospital infection prevention committee designed screening questions to identify patients at high risk for potential SARS-CoV-2 infection. Screening questions were based on CDC guidance and information available through the state division of public health for essential services screening [15,16]. Although CDC recommendations were updated regularly, screening and triage recommendations were largely unchanged during the study period. During the study period, the following three CSP questions were asked of all patients and their accompanying caregivers at ED triage: 1. Within the last 10 days, have you experienced a loss of taste or smell, persistent cough, shortness of breath, sore throat, or fever (<18 years of age ≥100.4°F; ≥18 years of age ≥99.5°F)? 2. Within the last 10 days, have you tested positive for COVID-19 or have a pending test related to symptoms or exposure? 3. Within the last 14 days, have you been near someone sick with known or suspected COVID-19?

Questions were posed directly to the patient when developmentally appropriate. In instances when a child was not able to directly answer the CSP questions, the child’s caregiver served as a surrogate respondent, answering CSP questions based on their observation of the child’s symptoms. If the patient or accompanying caregiver answered “yes” to any of the above questions, the patient was deemed CSP+. Patients were designated CSP- only if answering “no” to all the above questions. Providers were informed of the patient’s CSP designation; patients received routine standard of care while in the ED. For patients deemed stable for discharge from the ED, the decision to obtain SARS-CoV-2 testing was made independently by the provider based on the patient’s symptoms and their potential COVID-19 exposures; this was largely guided by the patient’s CSP designation. All patients being admitted to the hospital or the OR received SARS-CoV-2 testing prior to admission.

CSP questions were asked of all patients presenting to the ED during the study period. Due to testing limitations at the peak of the pandemic, SARS-CoV-2 testing was not able to be completed on all patients. Patients with SARS-CoV-2 testing completed as a requirement for hospital admission or at the discretion of the ED provider were ultimately included in the study. Patients over 21 years of age and those without SARS-CoV-2 testing were excluded. Regarding SARS-CoV-2 testing, four different tests were utilized: rapid polymerase chain reaction (PCR) (Xpert Xpress SARS-CoV-2/Flu/RSV), respiratory viral panel PCR (BioFire Respiratory Panel 2.1), standard immunology lab (CDC 2019-Novel Coronavirus Real-Time RT-PCR Diagnostic Panel), and commercially available real-time reverse transcriptase (rRT) PCR testing. 

Data collection and data analysis

Patients were identified retrospectively using the previously mentioned internal database. The CSP designation, SARS-CoV-2 test result, and disposition were recorded from this database. Demographic information including patient sex, age, race, and ethnicity was obtained from the hospital’s electronic data warehouse.

Sample size calculation to power the main outcome of the study, sensitivity, and negative predictive values of CSP were performed using pilot data. Data were analyzed using SPSS v.27 (IBM Corp, Armonk, NY). Descriptive statistics including means and standard deviations were calculated for continuous variables; counts and percentages were calculated for categorical variables. Statistical significance was determined using chi-square or a comparison of means where appropriate; statistical significance was determined to be p<0.05. Sensitivity, specificity, negative predictive value (NPV), and positive predictive value (PPV) with 95% confidence intervals (CIs) were calculated based on the construction of two-by-two tables for each patient cohort.

## Results

During the study period, 5,937 patients were seen in our free-standing tertiary care PED, of whom 5,908 patients were ≤ 21 years of age. A total of 1,613 (27.3%) patients ≤ 21 years of age had SARS-CoV-2 testing completed and were included in the study sample. The demographics of the study population undergoing SARS-CoV-2 testing were similar to those presenting during the study period, although some statistically significant differences did exist (Table 1). The study population was 51.0% (n=822) male, 47.8% (n=771) Caucasian, 37.2% (n=600) African American, 82.6% (n=1,4332) non-Hispanic/Latino, 16.7% (n=269) Hispanic or Latino, and the mean age was 6.9 (± 6.2) years.

**Table 1:** Demographics of patients ≤ 21 years old presenting during the study period who were SARS-CoV-2 tested (n=1,613) and those who were not tested (n=4,295) SARS-CoV-2: severe acute respiratory syndrome coronavirus 2.

	SARS-CoV-2 Tested (n=1,613)	Not SARS-CoV-2 Tested (n=4,295)	P Value
Age (years)	Mean= 6.9±6.2	Mean=7.3±5.9	<0.001>
Sex (%)			
Male	822 (51.0)	2,183 (50.8)	
Female	791 (49.0)	2,112 (49.2)	0.930
Race (%)			
Caucasian	771 (47.8)	2,240 (52.2)	0.002
African American/Black	600 (37.2)	1,390 (32.4)	
Asian	21 (1.3)	70 (1.6)	
American Indian or Alaska Native	5 (0.3)	6 (0.1)	
Ethnicity (%)			
Non-Hispanic/Latino	1,332 (82.6)	3,498 (81.4)	<0.207
Hispanic or Latino	269 (16.7)	780 (18.2)	

Of the 1,613 patients with SARS-CoV-2 testing completed during the study period, 147 patients (9.1%) tested positive for SARS-CoV-2; their demographics are listed in Table 2. The mean age in patients with positive tests was 8 (±6.7) years. Significant differences in sex and ethnicity were noted with regard to test positivity with a higher proportion of positive tests in female patients (p=0.036). There was no difference in the positivity rate between whites and African Americans (p=0.089).

**Table 2:** Demographics of COVID-positive (n=147) and COVID-negative patients (n=1,466) ^a^No significant difference existed in SARS-CoV-2 test positivity between Caucasian and African American/Black patients. SARS-CoV-2: severe acute respiratory syndrome coronavirus 2.

	COVID-19-Positive Only (n=147, 9.1%)	COVID-19-Negative Only (n=1,466, 90.9%)	P Value
Age (years)	8.0 ± 6.7	6.9 ± 6.1	
Sex (%)			
Male	62 (42.2)	760 (51.8)	0.025
Female	85 (57.8)	706 (48.2)	
Race (%)			
Caucasian	56 (38.1)	715 (48.8)	0.089^a^
African American/Black	59 (40.1)	541 (36.9)	
Asian	3 (2.0)	18 (1.2)	
American Indian or Alaska Native	0 (0.0)	5 (0.3)	
Some other races/refused	29 (19.7)	187 (12.8)	
Ethnicity (%)			
Non-Hispanic/Latino	111 (75.5)	1,221 (83.3)	0.036
Hispanic or Latino	34 (23.1)	237 (16.2)	

Overall, 1,014 (62.9%) of tested patients were CSP+ with 131 (12.9%) of these patients ultimately testing positive for SARS-CoV-2. Comparatively, 599 (37.1%) patients were CSP- with only 16 (2.7%) positive tests for SARS-CoV-2 infection in the CSP- group. The test positivity rate between the CSP+ and CSP- groups was significant (p<0.0001). The sensitivity of the CSP was 89.1%, while the specificity of the test was only 39.8%. Two-sided 95% CIs for sensitivity and specificity are 82.9%-93.7% and 37.3%-42.3%, respectively. The NPV of the CSP was 97.3% (95% CI=95.8%-98.3%); however, the PPV of the screen was only 13.0% (95% CI=12.2%-13.7%) (Table 3).

**Table 3:** Sensitivity, specificity, NPV, and PPV for COVID-19 screening protocol based on patient cohort CI, confidence interval; OR, operating room; NPV, negative predictive value; PPV, positive predictive value.

Patient Cohort	Sensitivity % (95% CI)	Specificity % (95% CI)	NPV (95% CI)	PPV (95% CI)
All tested patients (n=1,613)	89.1 (82.9-93.7)	39.8 (37.3-42.3)	97.3 (95.8-98.3)	12.9 (12.2-13.7)
All admitted patients (n=887)	66.7 (49.8-80.9)	63.3 (60.0-66.6)	97.6 (96.4-98.5)	7.7 (6.2-9.6)
Subset admitted to OR (n=149)	16.7 (0.4-64.1)	83.9 (76.9-89.5)	96.0 (94.3-97.2)	4.2 (0.7-21.3)
Discharged patients (n=726)	97.2 (92.1-99.4)	7.4 (5.5-9.8)	93.9 (82.9-98.0)	15.5 (15.0-16.0)

**Figure 1:**
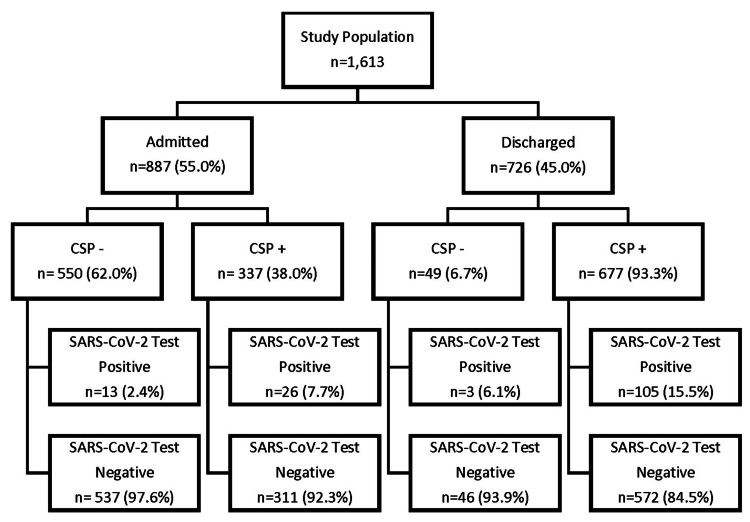
Summary of SARS-CoV-2 test results for admitted and discharged patients based on CSP designation CSP-: COVID-19 screening protocol negative, CSP+: COVID-19 screening protocol positive, SARS-CoV-2: severe acute respiratory syndrome coronavirus 2.

Of the tested patients, 887 (55.0%) were admitted to the hospital and 726 (45.0%) were discharged from the PED (Figure 1). All patients admitted to the hospital received SARS-CoV-2 testing. Within the subset of the admitted patients, there was a significant difference (p=0.001) in the rate of positive SARS-CoV-2 tests in the CSP+ (7.7%) versus CSP- (2.4%) groups. In the admitted group, the sensitivity of the CSP was 66.7% (95% CI=49.5%-80.9%) and the specificity of the CSP was 63.3% (95% CI=60.0%-66.6%). The NPV of the CSP in admitted patients was 97.6% (95% CI=96.4%-98.5%); the PPV of the screen was only 7.7% (95% CI=6.2%-9.6%) (Table 3).

Of the 887 admitted individuals, 738 (83.2%) were admitted to the wards and 149 (16.8%) patients were directly admitted to the OR. Of the patients admitted to the OR, 83.9% (n=125) had negative COVID-19 screens with 4.0% (n=5) testing positive for SARS-CoV-2. There was no statistically significant difference (p=0.97) in positive SARS-CoV-2 test results in patients admitted to the OR with negative versus positive COVID-19 screens. In patients admitted directly to the OR, the sensitivity of the CSP was only 16.7% (95% CI=0.4%-64.1%), while the specificity of the CSP was 83.9% (95% CI=76.9%-89.5%). The NPV of the CSP patients admitted to the OR was 96.0% (95% CI=94.3%-97.2%); the PPV of the screen was only 4.2% (95% CI=0.7%-21.3%) (Table 3).

Of the 726 patients discharged from the ED, 14.9% (n=108) tested positive. In patients discharged from the ED, the sensitivity of the CSP was 97.2% (95% CI=92.1%-99.4%), while the specificity of the CSP was 7.4% (95% CI=5.5%-9.8%). The NPV of the CSP in discharged patients was 93.8% (95% CI=82.9%-98.0%); the PPV of the screen was 15.5% (95% CI=15.0%-16.0%) (Table 3). Among the discharged patients, there was not a significant difference in the rate of positive tests between those who screened positive and negative (p=0.094).

## Discussion

This study demonstrates a significant difference in the rate of positive SARS-CoV-2 tests in patients screened for COVID-19 infection in the early phases of the pandemic. This statistical significance was demonstrated between the CSP+ and CSP- groups in patients admitted to the OR and discharged from the ED; the NPV of the screen was above 93% in both groups making a negative screen a reliable predictor of the absence of COVID-19 infection.

At our institution, the CSP was initially used to enable patient cohorting and continues to be used to guide PPE precautions for healthcare workers. This is a method adopted by many free-standing children’s hospitals around the world at the start of the pandemic. Several international groups have published reports regarding adaptations made to the PED during the pandemic to cohort asymptomatic patients from those deemed high risk of potential SARS-CoV-2 infection [3,4]. However, these reports simply outline adaptations made in the PED and do not attempt to determine the effectiveness of the screening process.

A study of adult patients presenting to an ED in Singapore revealed an 84.3% sensitivity of an internal screening protocol based on clinical symptoms and epidemiologic risk factors [5]. Yet, screening for COVID-19 infection in the pediatric patient population has proven to be challenging given lower rates of infection and increased frequency of asymptomatic infections. This is demonstrated by an Italian study completed at the start of the pandemic in which SARS-CoV-2 testing was performed on all children admitted to the hospital with no confirmed COVID-19 cases despite one-third of patients screening at risk for suspected infection [17]. However, some groups have had increased success in investigating the relationship between screening and SARS-CoV-2 test positivity in pediatrics.

When comparing COVID-19 patient characteristics in Turkey, Akca et al. reported that 93.7% of pediatric patients with COVID-19 had positive household contact and 83.2% were symptomatic in the early months of the pandemic [18]. Similarly, a study completed by Stacevičienė et al. during the first three months of the pandemic evaluated the use of the COVID-19 screening process in Lithuania noting nearly all SARS-CoV-2-positive patients both symptomatic and had exposure to the virus [19]. In a follow-up publication, the group expanded data collection to include the first full year of the pandemic (March 2020 to February 2021). The results continued to demonstrate a predominance of positive tests occurring in patients with a history of COVID-19 exposure and symptoms but noted that SARS-CoV-2 infection was identified in patients without these risk factors during later phases of the pandemic [20]. The results of the latter align with our study’s findings noting a small proportion of positive SARS-CoV-2 tests, approximately 10% (16/147), resulting from the CSP- group. This is likely due to the timing of our study (November 2020 to December 2020), which overlapped with the third phase (October 2020 to February 2021) of the Lithuanian investigation during which they demonstrated a significantly higher number of SARS-CoV-2 tests [20].

Yet, despite positive cases of COVID-19 detected in the CSP- group, this study does demonstrate a high NPV of the screen across all investigated groups. This is supported by the work of Roversi et al. who found that 99.3% of pediatric patients tested negative for SARS-CoV-2 when they had no reported exposure and no fever [21]. In contrast, the PPV of the screen was found to be low across all patient cohorts in this study. The low PPV of the screen reinforces the notion that it remains difficult to distinguish COVID-19 infection in children from other viral illnesses. An Austrian study evaluating the question determined that aside from having a temperature greater than 37.5°C, symptoms at the time of presentation did not differ significantly between patients with and without COVID-19 [22].

Given that this study was completed at one of the peaks of the pandemic, it was not possible to test all patients presenting to the ED due to resource limitations and the lack of clinical necessity. All patients admitted to the hospital received COVID-19 testing to ensure proper isolation precautions within the hospital system. This universal testing of admitted patients helps to eliminate a degree of selection bias and may be more reflective of the general population. When looking at the results for the subset of admitted patients, there is a significant difference in SARS-CoV-2 test positivity in patients admitted from the CSP+ versus the CSP- groups. This has important implications not only for patient cohorting and PPE guidance in the hospital setting but also for the outpatient setting where screening questionnaires are used routinely to risk stratify patients presenting to the office.

This study highlights the importance of COVID testing prior to admission to the OR because the CSP was not able to significantly discriminate possible COVID-19 infection in this cohort. This is especially important given the invasive nature of surgical procedures and the greater potential for disease transmission in the OR [23]. The lack of statistical significance in this group may be the result of sample size limitations with very few patients being admitted to the OR from the CSP+ group. Nonetheless, the CSP did work for most patients admitted to the OR with only one testing positive for SARS-CoV-2 in the CSP- group.

Regarding the CSP, the cohort of patients discharged from the ED, the CSP was not able to discriminate SARS-CoV-2 test positivity in the CSP+ and CSP- groups. The study was not powered for this analysis making it difficult to make conclusions about this population at this time. Yet, the CSP did have a sensitivity of 97.2% in the discharged patient population suggesting that the use of a symptom- and exposure-based questionnaire may be useful to guide testing especially when resources are limited.

As previously discussed, the possibility of selection bias is a limitation of this study. Except for patients being admitted to the hospital, the decision to obtain SARS-CoV-2 testing was at the discretion of the ED provider based on the patient’s symptoms and exposure history. It is likely that asymptomatic patients or those with atypical symptoms who were discharged were not captured in this analysis. This study is also limited by the inclusion of only patients with SARS-CoV-2 testing; diagnoses and CSP designations for patients without testing completed were not able to be examined in this analysis. Additional limitations include that the study was conducted at a single center and performed retrospectively. Furthermore, this study was completed in a tertiary care pediatric center making it difficult to generalize results to the adult patient population for use in the general ED. Additional prospective research with randomized testing of asymptomatic patients or universal testing of all patients is needed to evaluate this further. Currently, screening is only an adjunctive measure in the effort to mitigate the spread of COVID-19; testing remains the mainstay to identify true SARS-CoV-2 infections.

This study was completed in the early phases of the pandemic, and ongoing research is needed to determine the efficacy of a CSP in the era of new SARS-CoV-2 variants. Additional studies are needed to understand the utility of COVID screening with the increased transmissibility of the SARS-CoV-2 Omicron variant [24-26]. Additionally, with COVID-19 vaccination approved in pediatric patients six months and older [27], considering vaccination status as a screening question should be investigated further.

## Conclusions

A symptom- and exposure-based COVID screening protocol designed using CDC guidance for SARS-CoV-2 infection is a reliable tool to direct testing in the pediatric population. The CSP used in this study has a high NPV across all patient cohorts with a low probability of disease in those with negative screens; this is particularly useful to direct PPE practices in the healthcare setting and for infection prevention purposes. The negative predictive values of screening in the pediatric population support its use during the initial phases of the COVID-19 pandemic and may prove to be useful in the emergence of new strains. The use of screening protocols may be particularly valuable in areas with limited resources or the setting of testing scarcity; however, screening is not a replacement for the gold-standard PCR testing for the detection of SARS-CoV-2 infection.
